# Effects of Prediabetes on Bone Metabolism and Healing in Orthognathic Surgery: A Narrative Review

**DOI:** 10.1055/s-0046-1820502

**Published:** 2026-07-08

**Authors:** Suchanon Siriwechadaruk, Warit Powcharoen, Kanich Tripuwabhrut, Napatsorn Imerb

**Affiliations:** 1 Department of Oral and Maxillofacial Surgery, Faculty of Dentistry, Chiang Mai University, Chiang Mai, Thailand; 2 Department of Orthodontics and Pediatric Dentistry, Faculty of Dentistry, Chiang Mai University, Muang, Chiang Mai, Thailand

**Keywords:** prediabetes, bone metabolic markers, systemic acceleratory phenomenon, orthognathic surgery

## Abstract

Prediabetes, defined by impaired glucose regulation, is an increasingly prevalent metabolic disorder with implications beyond glycemic control. Its impact on bone remodeling and healing is gaining attention, particularly in orthognathic surgery. This narrative review aimed to map the current evidence regarding the impact of prediabetes on bone healing, metabolic markers, and surgical outcomes in orthognathic surgery. A comprehensive literature search was performed (PubMed, Scopus, Web of Science, Cochrane Library, and Google Scholar) up to January 2025, yielding 32 eligible studies. Emerging data suggest that prediabetes may influence bone homeostasis through insulin resistance, low-grade chronic inflammation, and the accumulation of advanced glycation end products. These changes may attenuate the systemic acceleratory phenomenon observed after surgical trauma, potentially delaying bone healing. However, current evidence is limited by significant assay heterogeneity, varying temporal sampling windows, and a lack of prospective surgical trials. While not as severe as overt diabetes, prediabetes constitutes an underrecognized but important risk factor in the perioperative management of orthognathic surgery patients. Future research should prioritize standardized prospective trials to establish evidence-based guidelines.

## Introduction

High blood glucose is a metabolic condition characterized by blood glucose levels exceeding the normal range. It includes both diabetes and nondiabetic hyperglycemia (prediabetes). While it is widely recognized that diabetes refers to a state where blood sugar levels are high enough to cause health complications, prediabetes is often overlooked because it usually presents no obvious symptoms. However, numerous recent studies have found that having elevated blood glucose levels, even if not high enough for a diabetes diagnosis. 1 It is often a precursor to type 2 diabetes and signals underlying systemic metabolic dysfunction. 1 Importantly, it affects a significant proportion of the global population, with the prevalence of impaired glucose tolerance (IGT) estimated at 9.1% (464 million adults) and impaired fasting glucose (IFG) at 5.8% (298 million adults) as of 2021. These figures are projected to rise to 10.0% (638 million) for IGT and 6.5% (414 million) for IFG by 2045, reflecting a growing public health challenge. 2 Although traditionally viewed in the context of diabetes progression and cardiovascular risk, prediabetes has significant implications for bone health. 3 4 Studies have shown that while bone mineral density (BMD) in individuals with prediabetes may appear normal or even elevated, bone quality and mechanical strength are often compromised. 5 6 These changes are attributed to the accumulation of advanced glycation end products (AGEs), chronic inflammation, and altered osteoblast and osteoclast activity, which disrupt normal bone remodeling processes. 7

Bone remodeling is a critical physiological process that maintains skeletal integrity and facilitates healing after injuries or surgeries. It involves a tightly regulated balance between bone formation by osteoblasts and bone resorption by osteoclasts. This balance could potentially be disrupted in systemic conditions like prediabetes, leading to delayed healing, increased fracture risk, and reduced bone strength. Such disruptions become particularly relevant in procedures like orthognathic surgery, which relies heavily on effective bone healing for successful outcomes. 8 Orthognathic surgery is a widely performed procedure aimed at correcting skeletal deformities and malocclusions in the maxillofacial region. The surgery often involves intricate osteotomies and the precise repositioning of jaw segments. 9 In contemporary practice, the integration of virtual surgical planning and advanced reconstruction workflows has become vital, as surgical precision directly influences the magnitude of trauma and subsequent healing dynamics. 10 11 The success of this procedure depends heavily on postsurgical bone healing, which stabilizes the repositioned skeletal segments, restores functional occlusion, and ensures optimal aesthetic and structural results. 9 Delays or impairments in bone healing can lead to complications such as nonunion, malunion, or increased risk of infection, jeopardizing the overall success of the surgery. 8

Notably, prediabetes is often overlooked, as it typically presents without noticeable symptoms, unlike type 2 diabetes; however, this silent condition can act as a critical, hidden factor contributing to the failure of certain treatments or surgical outcomes, making it a potentially underestimated risk in clinical practice. In the context of prediabetes, impaired glucose metabolism and associated factors such as chronic inflammation, elevated AGEs, and reduced angiogenesis can negatively influence the bone healing process. 7 This underscores the importance of glycemic control as a critical factor in determining the success of orthognathic surgery. Maintaining stable blood glucose levels can potentially mitigate the adverse effects of prediabetes on bone remodeling, facilitating better surgical outcomes and reducing the risk of complications. Thus, integrating metabolic management into the perioperative care of patients undergoing orthognathic surgery is vital for optimizing bone healing and achieving long-term success.

Additionally, prediabetes adds an additional layer of complexity to bone healing in orthognathic surgery. The systemic effects of prediabetes, including altered glucose metabolism, reduced angiogenesis, and impaired collagen synthesis, can significantly impair the healing process. 12 Furthermore, SAP, a response triggered by surgical trauma, may behave differently in individuals with metabolic disturbance. 13 While SAP generally facilitates accelerated healing, in prediabetic patients, the response may be maladaptive, potentially resulting in suboptimal healing outcomes. Although direct studies investigating the behavior of SAP in prediabetic individuals are limited, it is highly plausible that the systemic metabolic disturbances characteristic of prediabetes could compromise the efficacy of SAP, underscoring the need for further research in this area.

This review provides an analysis of the impact of prediabetes on bone metabolic markers and healing mechanisms, with a particular focus on orthognathic surgery. It synthesizes current evidence on the pathophysiological mechanisms underlying prediabetes-related alterations in bone remodeling and highlights key metabolic markers such as CTX-I, osteocalcin, sclerostin, and BAP, which are crucial for assessing bone healing, the influence of dysglycemia on orthognathic surgery outcomes, and evidence-based clinical management strategies. Furthermore, this review identifies gaps in the current literature and provides insights to guide future research aimed at optimizing surgical outcomes for prediabetic patients undergoing orthognathic procedures.

## Methods

This study was conducted as a narrative literature review supported by a structured search strategy to summarize current evidence regarding the impact of prediabetes on bone metabolic markers and bone healing in the context of orthognathic surgery. As the study analyzed previously published literature and did not involve human participants or identifiable patient data, institutional review board approval was not required. The review process was guided by the Scale for the Assessment of Narrative Review Articles (SANRA) criteria, 14 which evaluates narrative reviews across six domains: relevance of the article, clarity of aims, description of the literature search, appropriate referencing, scientific reasoning, and presentation of data. In addition, the reporting structure was designed to follow transparent reporting principles inspired by PRISMA, including the use of a flow diagram to illustrate the study identification and selection process.

A structured literature search was independently performed by two reviewers (S.S. and N.I.) using the electronic databases PubMed, Scopus, and Web of Science (WoS). The search incorporated the following keywords: ((Prediabetes OR bone healing OR bone metabolic marker OR systemic acceleratory phenomenon) AND orthognathic surgery). The search syntax was adapted as necessary for each database to optimize the retrieval of relevant studies. In addition, the reference lists of selected articles were manually screened to identify additional relevant publications that might not have been captured through the database search. Titles and abstracts retrieved from database searches were independently screened, and articles considered relevant during the initial screening stage were subsequently subjected to full-text assessment to determine eligibility.

Studies were included if they investigated bone metabolic markers, bone remodeling, or bone healing in relation to impaired glucose metabolism, including prediabetes, IFG, IGT, or insulin resistance. Studies addressing orthognathic surgery or bone healing following jaw surgery were considered eligible. Only original research articles published in English were included, and studies were excluded if they were case reports, review articles, editorials, or conference abstracts, or if they were unrelated to bone metabolism or glucose dysregulation. Because the number of studies directly examining prediabetes in patients undergoing orthognathic surgery remains limited, studies evaluating bone metabolic responses to surgical trauma or metabolic dysregulation in other skeletal contexts were also considered. These studies were included to provide relevant mechanistic insights into the relationship between systemic metabolic disturbances and bone healing processes.

The findings from eligible studies were qualitatively synthesized, and the evidence was organized into thematic categories. These categories included the dynamics of bone metabolic markers following orthognathic surgery, the systemic effects of prediabetes on bone metabolism and remodeling, and therapeutic strategies aimed at improving bone homeostasis and bone healing under conditions of impaired glucose metabolism.

## Overview of Study Selection and Characteristics

The study selection process, as illustrated in Fig. 1 , began with an initial database search that identified 265 potential studies. After removing 32 duplicate records, 233 unique studies remained for title and abstract screening. Of these, 164 studies were excluded due to irrelevance, lack of focus on bone metabolic markers, or inadequate assessment of prediabetes-related bone changes. The remaining 69 studies underwent full-text evaluation, resulting in the exclusion of 37 studies that did not meet the predefined inclusion criteria. These exclusions were primarily due to insufficient data on orthognathic surgery or a lack of comprehensive bone marker analysis. A total of 32 studies were included in the qualitative synthesis. Detailed characteristics, including assay methods (e.g., ELISA, ECLIA), are summarized in Table 1 . Due to the significant methodological heterogeneity observed across the included studies (particularly in the use of varying assay techniques and differing temporal sampling windows), a qualitative rather than quantitative synthesis was performed. Heterogeneity was addressed by categorizing studies based on their physiological focus and appraising the level of evidence for each study to ensure a balanced interpretation of conflicting biochemical results.

**Fig. 1:**
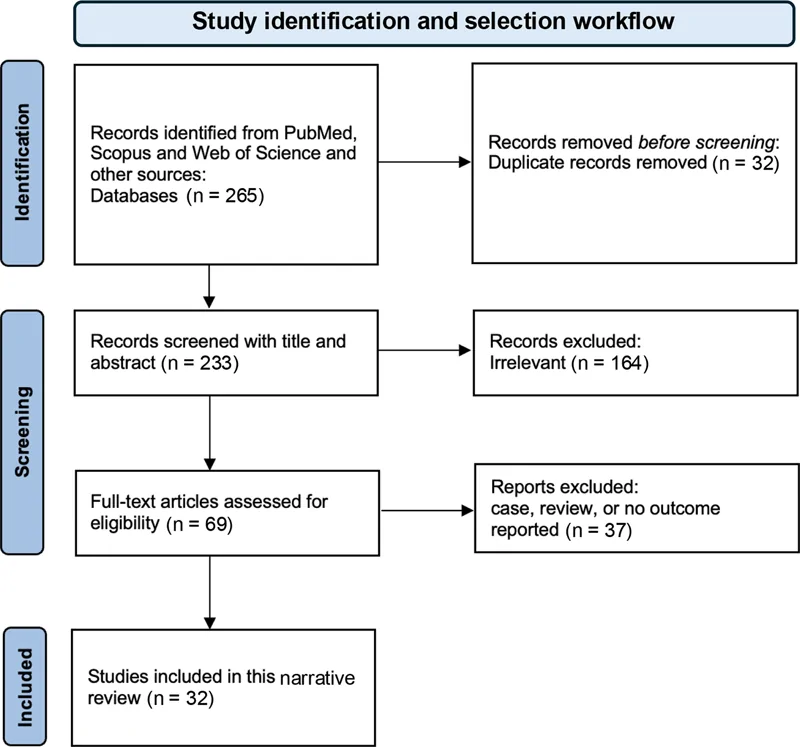
Flow diagram of the literature search and study selection process.

**Table 1:** Comprehensive characteristics and methodological summary of included studies

No.	Author (year)	Study design	Sample size ( *N* )	Intervention and population	Primary outcomes	Targeted bone markers	Assay platform and specific methodology	Reported units
1	Miyaoka et al (2005) 20	Prospective cohort	21	Mandibular prognathism (BSSRO)	IL-6, IL-10, CRP, and neutrophil	- IL-6, IL-10 - CRP	- Sandwich ELISA - ACCP/SCCM criteria for SIRS	- pg/mL - mg/L
2	Liou et al (2011) 19	Prospective cohort	22	Le Fort I and BSSRO (Surgical First)	ICTP, ALP, OTM	- ICTP - ALP	- Standard laboratory methods (not specified)	- ng/mL
3	Yang et al (2012) 22	Prospective cohort	19	Orthognathic surgery patients	OCN+ cells, IGF-I levels OCN, ALP, and COL-1 (collagen type 1) gene expression	- OCN+ cells - IGF-I	- Flow cytometry and Immunocytochemical staining - ELISA (Boster, Inc., Wuhan, China, ELISA kit)	- % - ng/mL
4	Soliman et al (2016) 24	Prospective cohort	9	Le Fort I and BSSRO	OPG	- OPG	- Sandwich ELISA	ng/mL
5	Huang et al (2019) 21	Experimental study	15 (H) / 18 (R)	BSSRO patients and Rabbit model	TRAP5b, BAP, bone volume	- TRAP5b, BAP	- Human ELISA kit (Abnova, Taipei, Taiwan)	–
6	Abe et al (2019) 13	Prospective cohort	28	Le Fort I and BSSRO	CRP, ICTP, ALP, BAP, OCN+ cell	- OCN+ cells - CRP - ICTP - BAP - ALP	- Flow cytometry - Latex agglomeration method (N-assay LA CRP-S Nittobo D-Type, Nittobo Medical Co., Ltd., Tokyo, Japan) - RIA2 antibody assay, Pirijinorin ICTP (Fujirebio, Inc., Gent, Belgium) - Chemiluminescent enzyme immunoassay (CLEIA) (Access Ostase, Beckman Coulter, Brea, CA, USA) - Colorimetric method (L-type Wako ALP J2, Wako Pure Chemical Industries, Ltd., Osaka, Japan)	- % - mg/dL - ng/mL - μg/L - U/L
7	Soliman et al (2019) 23	Prospective cohort	9	Le Fort I and BSSRO	TGF-β1, OPG, cytokines	- TGF-β1, OPG	- ELISA	- ng/mL
8	Kaida et al (2008) 17	Prospective cohort	16	Skeletal Class III (BSSRO)	OCN, BAP, ALP, TRAP, CRP	- OCN - BAP - ALP, TRAP - CRP	- RIA (YAMASA” RIA kit, Chiba, Japan) - RIA (ALKPHASE-B kit, Metra Biosystems Inc., Mountain View, CA, USA) - EDTA-titration for serum Ca determination, photometry - Standard laboratory methods (not specified)	- ng/mL - U/L - IU/L - mg/dL
9	Hakobyan et al (2024) 25	Prospective cohort	48	Mandibulectomy and fibula flap	OCN, β-Cross-Laps, BMD	- OCN, β-cross-Laps	ECLIA (IFA ELISA, Denmark)	ng/mL
10	Wozniak et al (2021) 28	Case–control	35	Mandibular fracture vs. control	NO, MDA, ADMA (oxidative)	–	–	–
11	Daniela et al (2012) 26	Case–control	58	Bone fracture vs. soft tissue	BAP, β-CTX, OCN	- BAP - β-CTX - OCN	- Enzymatic bone ALP-test, Roche Diagnostics (Mannheim, Germany) - One-step sandwich ELISA (Immunologic β-CrossLaps/serum-test, Roche Diagnostics, Mannheim, Germany) - One-step sandwich ELISA, Immunologic N-MID Osteocalcin-test (Roche Diagnostics, Mannheim, Germany)	- U/L - ng/mL - ng/mL
12	Kenanidis et al (2010) 27	Prospective cohort	57	Postmenopausal	OPG, RANKL, OCN, BAP	- OPG - RANK - OCN - BAP	- Sandwich ELISA - EIA - Radioimmunoassay - EIA	- pmol/l - pmol/l - ng/mL - mg/L
13	Liu et al (2017) 33	Longitudinal cohort	195	NGT progressing to dysglycemia	Serum CTX	CTX	ECLIA (Elecsys β-Crosslaps/serum, Cobas 601, Roche Diagnostics)	ng/mL
14	Rahimi-Nedjat (2017) 8	Retrospective	1,374	Maxillofacial surgery patients	Hyperglycemia and infection risk	–	–	–
15	Holloway-Kew (2019) 41	Cross-sectional	2,113	IFG/T2DM (BMD assessment)	CTX, P1NP, femoral BMD	- CTX - P1NP	-ECLIA (Automated Roche Modular Analytics E170 Analyser, Roche Diagnostics, Mannheim, Germany)	- ng/mL - μg/L
16	Daniele et al (2015) 4	Case–control	122	Prediabetes (OGTT/clamp)	Sclerostin, insulin sensitivity	- Sclerostin	- ELISA (Biomedica, Vienna, Austria)	- pmol/L
17	Duan et al (2017) 40	Case–control	271	Postmenopausal prediabetes/T2DM	OPG, HOMA-IR, spinal BMD	- OPG	- ELISA (RayBiotech, Norcross, GA, USA)	- pg/mL
18	Ojala et al (2020) 42	RCT	54	Insulin resistant (SIT vs. MICT)	Bone marrow glucose uptake	–	–	–
19	Akyay et al (2023) 46	RCT	30	Postmenopausal (exenatide/insulin)	RANK, RANKL, OPG, ALP, BAP, BMD	- OPG, RANK, RANKL - ALP, BAP	- ELISA (Elabscience) - ELISA (MicroVue human ELISA kit)	- ng/mL - μg/L
20	Stage et al (2018) 47	RCT	371	T2DM (insulin ± metformin)	CTX, P1NP, HbA1c control	- CTX - P1NP	- Chemiluminescence method (iSYS, Immunodiagnostic Systems Ltd., Boldon, England)	- ng/mL - μg/L
21	Zinman et al (2010) 48	RCT (ADOPT)	1,605	T2DM (monotherapy trial)	CTX, P1NP, BAP, estradiol	- CTX - P1NP - BAP	- ECLIA (Nordic Biosciences, Copenhagen, Denmark) - RIA (Orion Diagnostica, Espoo, Finland) - Chemiluminescence (Beckman Access (Fullerton, CA))	- ng/mL - μg/L - μg/L
22	Wang et al (2020) 49	RCT	120	Elderly T2DM (metformin dose)	BMD, OCN, β-CTX	- OCN - β-CTX	-ECLIA (Infinite F50 ELISA reader)	ng/mL
23	Jensen et al (2024) 50	RCT	195	Obese (liraglutide ± exercise)	BMD, CTX, P1NP, lean mass	- CTX-1 - P1NP	- Chemiluminescence method (iSYS CTX, Immunodiagnostic Systems) - Chemiluminescence method (iSYS intact serum procollagen type I N-propeptide assays, Immunodiagnostic Systems)	- ng/L - μg/L
24	Rajabi-Naeeni (2020) 51	RCT	168	Prediabetes (omega-3 + vitamin D)	FBS, HOMA-IR, HOMA-B	–	–	–
25	Frost et al (2018) 3	Prospective and cross-sectional (RISC study)	576	Nondiabetic men (RISC cohort)	- P1NP - CTX - Insulin sensitivity - Insulin secretion	- CTX, P1NP	- Chemiluminescence method (IDS-iSYS)	- μg/L
26	Iglesias et al (2011) 16	Case–control	64	Obese subjects with NGT, prediabetes, and T2D	- Glucose metabolism - OCN - P1NP - β-CTX	- OCN - P1NP, β-CTX	ECLIA (Elecsys 2010, Roche Diagnostics, Basel, Switzerland)	- nmol/l - μg/L
27	Pittas et al (2009) 55	Cross-sectional	380	Older adults assessing metabolic phenotype and bone markers	- Glucose metabolism - OCN - uNTX	- OCN - uNTX	- Two-site IRMA (Nichols Institute, San Juan Capistrano, CA) - ELISA (Ostex International, Seattle, WA)	- ng/mL - nmol/mmol
28	Huang et al (2017) 35	Cross-sectional	118	Healthy volunteers	- TRAP - Metabolic syndrome (MetS) - IL-6 - CRP	- TRAP	- Sandwich ELISA	- μg/L
29	Gateva et al (2018) 36	Cross-sectional	80	Obese individuals with and without prediabetes	- FGF-23 - PTH - 25(OH)D - Klotho	- FGF23	- ELISA (MYBIOSOURCE)	- pg/mL
30	Cheung et al (2013) 37	Cross-sectional	3,773	Nondiabetic U.S. adults Insulin resistance by HOMA2-IR	- Glucose metabolism - ALP - BAP	- BAP - ALP	- ELISA (Hybritech Tandem-MP Ostase immunoenzymetric assay and chemiluminescence method (Beckman Access Ostase assay) - Hitachi model 704 multichannel Analyzer Spectrophotometry and Beckman Synchron LX20	- μg/L - U/L
31	Faramarzi et al (2025) 38	Cross-sectional (Azar cohort)	14,865	General population	- Glucose metabolism - ALP - ALT - AST - Gamma-glutamyl transferase	- ALP	- Pars Azmoon kit (Tehran, Iran)	- IU/L
32	Mashavi et al (2017) 56	Cross-sectional	114	Postmenopausal osteoporotic women with different glucose tolerance	- Glucose metabolism - OPG - BMD - CRP - BMD	- OPG	- ELISA (BioVendor, Asheville, NC)	- pmol/L

## Overview of Prediabetes and Its Systemic Effects

Prediabetes is defined as blood glucose levels elevated above normal but below the threshold for type 2 diabetes. It reflects impaired glucose metabolism, primarily due to insulin resistance and/or reduced insulin secretion and it is recognized as a strong predictor of diabetes progression. 1 The American Diabetes Association (ADA) defines prediabetes based on specific glycemic thresholds: a fasting plasma glucose (FPG) level of 100 to 125 mg/dL, a 2-hour oral glucose tolerance test (OGTT) result of 140 to 199 mg/dL, or an HbA1c level of 5.7 to 6.4%. 2 Numerous studies have indicated that the prevalence of prediabetes is continuously rising worldwide. However, the exact prevalence varies depending on the specific study and the region being examined. 2

Globally, prediabetes prevalence is increasing at an alarming rate, with hundreds of millions affected worldwide and projections indicating a continued rise by 2045. Although prevalence varies by region, age, and socioeconomic context, the upward trend is consistent, and younger populations are increasingly affected. 2 Beyond glycemic control, prediabetes has systemic implications. Chronic low-grade inflammation, vascular dysfunction, and metabolic dysregulation contribute to multiorgan effects, increasing risks of cardiovascular disease, impaired bone metabolism, and poor surgical outcomes. 15 These systemic alterations highlight the importance of early detection, preventive strategies, and targeted interventions to reduce both the progression to diabetes and the broader health consequences of prediabetes.

## Bone Metabolism and Relevance of Orthognathic Surgery in Bone Health Studies

Bone is the primary hard tissue in the body and it undergoes continuous changes through a dynamic process known as bone remodeling. This process comprises two key phases: bone formation and bone resorption. Bone formation involves the deposition of bone mass, a function performed by osteoblasts, while bone resorption entails the breakdown of bone mass, driven by osteoclast activity. Under normal conditions, these processes are tightly regulated and remain in balance to maintain skeletal integrity. However, certain conditions can disrupt this balance, leading to increased bone remodeling. These include physiological responses to bone fractures, postsurgical recovery, or the presence of systemic diseases. Such disruptions can impair bone remodeling and compromise bone health, as seen in conditions such as osteoporosis and various metabolic disorders. 6 8 9 Although direct measurement of bone structure or strength in living patients is challenging, several indirect methods provide valuable insights into bone metabolism and remodeling. These include the analysis of bone metabolic markers, such as growth factors, enzymes, hormones, and metabolic by-products, alongside assessments of bone radiographic density. 3 4 16 Together, these tools offer effective means to evaluate and understand bone remodeling processes in both health and disease.

Orthognathic surgery is a corrective procedure for addressing skeletal and occlusal deformities in the maxillofacial region. This surgery involves deliberate osteotomies to reposition the maxilla, mandible, or both, requiring efficient bone healing and remodeling to stabilize the repositioned segments and ensure successful functional and aesthetic outcomes. Given the intricate and localized nature of the bone remodeling process after orthognathic surgery, it provides an invaluable clinical setting for exploring the dynamics of bone metabolism. 13 Bone metabolism is crucial in the postoperative phase of orthognathic surgery, as it dictates the healing and integration of the repositioned skeletal segments. This process involves the coordinated activity of osteoclasts and osteoblasts to resorb damaged bone and deposit new bone matrix. 17 The balance between these activities is influenced by systemic and local factors, including growth factors, cytokines, mechanical stress, and patient-specific health conditions such as impaired glucose metabolism. Systemic conditions, particularly prediabetes, can impair this balance, resulting in delayed healing, reduced bone strength, and increased risk of complications like nonunion or infection. Complications such as nerve injury and wound problems are part of the risk profile that may be worsened by impaired bone healing. 18 The study of bone health in the context of orthognathic surgery is particularly relevant when considering the role of SAP and regional acceleratory phenomena (RAP). 19 Both processes enhance bone remodeling after surgical trauma, but their efficacy may be altered in individuals with systemic metabolic disturbances such as prediabetes. Understanding how conditions like prediabetes modulate SAP and RAP is essential for optimizing surgical outcomes and developing tailored perioperative care strategies.

Moreover, orthognathic surgery allows for the direct evaluation of bone metabolic markers, such as CTX-I, osteocalcin, and sclerostin, which reflect changes in bone turnover. 13 The temporal expression of these markers during the healing process provides critical insights into the dynamics of bone remodeling, both in healthy individuals and in patients with systemic conditions. For instance, studies have demonstrated that inflammatory cytokines (e.g., interleukin-6 [IL-6], C-reactive protein [CRP], and neutrophil activity) were observed shortly after surgery, emphasizing the acute inflammatory response to surgical trauma. 13 20 This response coincides with increased osteoclastic activity, reflected by the rise in bone resorption markers like C-terminal telopeptide of type I collagen(ICTP) and tartrate-resistant acid phosphatase (TRAP). 13 19 21 The heightened osteoclastic activity facilitates the removal of damaged bone tissue, an essential step in initiating bone remodeling. Following the initial resorption phase, markers of bone formation, including alkaline phosphatase (ALP), bone-specific alkaline phosphatase (BAP), and osteocalcin, increased gradually over weeks to months. 17 19 22 This delayed rise indicates the reparative phase of bone remodeling, where osteoblasts deposit new bone to replace resorbed tissue. The study on circulating osteocalcin-positive osteoblast lineage cells provides evidence of their mobilization and potential role in bone repair, further emphasizing the regenerative processes initiated by orthognathic surgery. 22 Additionally, the continuous increase in osteoprotegerin (OPG) and transforming growth factor-β 1 (TGF-β1) levels over several weeks 23 24 suggests their involvement in regulating bone remodeling. OPG acts as a decoy receptor for RANKL, inhibiting osteoclast activation and promoting bone formation, while TGF-β1 contributes to tissue regeneration and immune regulation. Across different interventions, a consistent sequence in bone remodeling emerges: initial suppression of bone formation markers (e.g., BAP, ALP), followed by an increase in bone resorption markers (e.g., β-CTX), and subsequent activation of bone formation markers (e.g., OCN). This sequence reflects the predictable phases of postoperative bone healing, with early resorption clearing damaged tissue and subsequent formation initiating bone regeneration. 25 26 27 The dynamics of bone turnover markers vary depending on the type of surgery and the bone involved. The comparison between hip and knee joint arthroplasty reveals distinct patterns in OPG, RANKL, and osteocalcin levels, likely due to differences in mechanical load, vascular supply, and the extent of surgical trauma. 27 Similarly, mandibulectomy with fibula-free flap reconstruction illustrates how extensive bone replacement influences the timeline of bone resorption and formation. 25 The interplay between systemic inflammatory and oxidative stress pathways and localized bone remodeling has also been highlighted in the context of jaw surgery. Elevated nitric oxide concentrations observed in mandibular fracture patients suggest their significant role in modulating both the inflammatory response and the reparative phases of bone healing. 28 Moreover, the correlation between specific marker levels and clinical outcomes, such as implant success or failure, underscores the importance of monitoring bone metabolic markers in surgical patients. For example, low OCN and high β-CTX levels were associated with implant failure in mandibulectomy patients. 25 This suggests that such markers could serve as early predictors of postoperative complications, enabling timely interventions. In addition, the temporal changes in bone markers provide valuable insights into the phases of healing. For instance, in joint arthroplasty, the biphasic increase in OPG and the delayed peak in BAP reflect distinct periods of bone remodeling and adaptation to mechanical stress. 27 These patterns can guide rehabilitation protocols and optimize the timeline of bone healing. Notably, differences in bone remodeling dynamics between cortical and trabecular bone are evident in the studies. For example, mandible fractures and femoral neck fractures exhibit unique marker profiles, likely due to variations in bone composition, vascularization, and biomechanical forces. 25 26 27 These findings highlight the need for site-specific approaches in managing bone healing.

While bone metabolic markers are widely utilized to evaluate bone turnover following orthognathic surgery, BMD offers complementary and valuable insights into the structural changes in bone metabolism that occur throughout the healing process. A study conducted by Lee et al showed that bone density significantly decreases at both alveolar and extra-alveolar sites following two-jaw orthognathic surgery. Using multidetector computed tomography (MDCT) and Hounsfield units (HU) as measures of density, the researchers observed reductions in both cancellous and cortical bone density, particularly in the mandibular and maxillary alveolar regions, as well as in systemic sites like the fourth cervical vertebra (C4). This decrease is attributed to RAP and SAP, which are biological responses that accelerate bone turnover and remodeling due to surgical trauma. 29 In a complementary study, Kang et al 30 examined bone evaluation through fractal analysis, which quantifies the complexity of trabecular bone in the mandible using panoramic radiographs. Their findings indicated a statistically significant decrease in fractal dimension (FD) 1 month after bilateral sagittal split ramus osteotomy, especially in the canine region. This reduction indicates transient osteopenia caused by RAP and the high metabolic turnover associated with healing bone. Additionally, On et al found that patients with osteopenia or osteoporosis—conditions characterized by reduced bone density—had significantly lower HU values at osteotomy sites in both the maxilla and mandible. This suggests that BMD can be influenced by underlying systemic bone conditions and that preoperative evaluation of HU values through CT may help predict complications such as unfavorable fractures or delayed bone healing. Notably, there was a strong positive correlation between T-scores from DEXA scans and HU values, confirming the usefulness of CT as a surrogate for assessing bone density in the maxillofacial region. 31

Collectively, these studies highlight the critical importance of understanding bone metabolism in the context of surgical interventions. The dynamic assessment of bone turnover markers offers a reliable, noninvasive approach for tracking the progression of bone healing, anticipating clinical outcomes, and guiding personalized patient care. The temporal patterns of these markers during the postoperative phase of orthognathic surgery are illustrated in Fig. 2 . A comprehensive overview of studies examining changes in bone metabolic markers and serum cytokines across various surgical sites is provided in Table 2 .

**Fig. 2:**
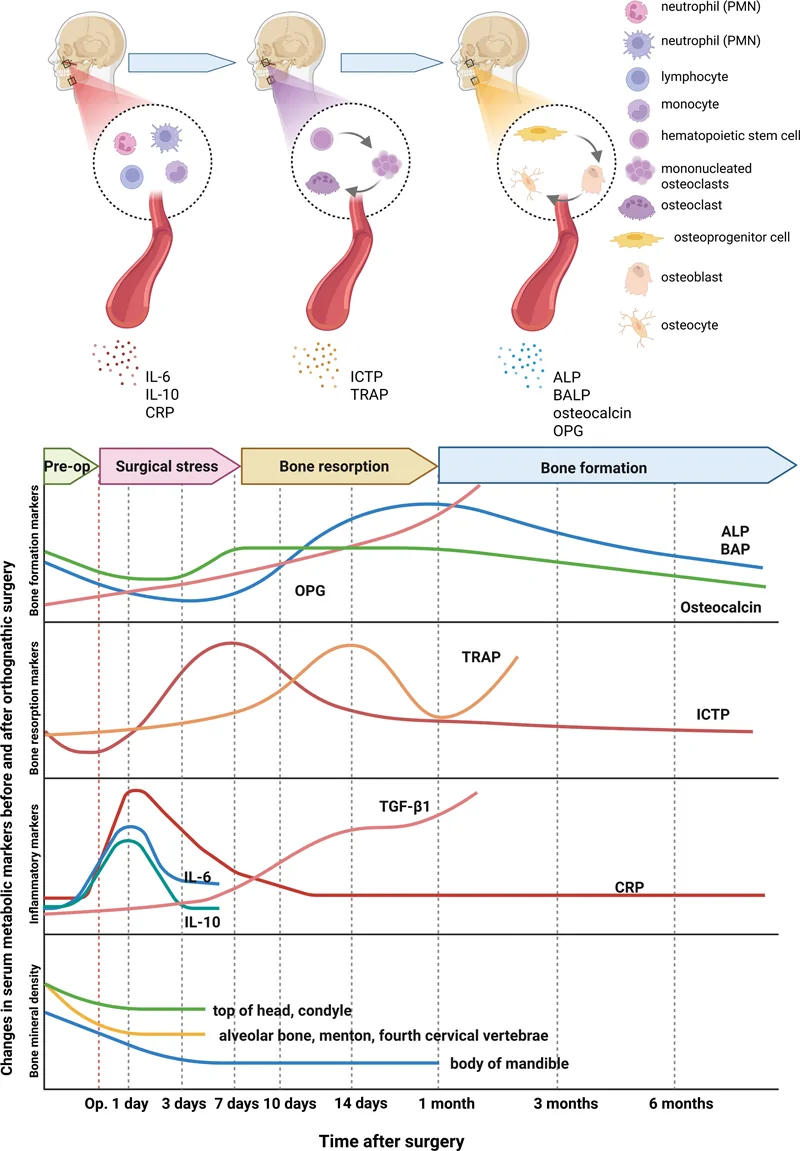
Dynamic changes in bone turnover markers and inflammatory response during the postoperative healing phase. The initial surgical stress phase triggers an acute inflammatory response characterized by elevated levels of IL-6, IL-10, and CRP, followed by a peak in bone resorption markers, including ICTP and TRAP. As bone resorption subsides, bone formation markers such as ALP, BAP, OCN, and OPG gradually increase, reflecting the regenerative phase. The graph also shows the progressive recovery of bone mineral density in different skeletal regions, indicating site-specific healing patterns. ALP, alkaline phosphatase; BAP, bone-specific alkaline phosphatase; BMD, bone mineral density; CRP, C-reactive protein, ICTP, C-terminal telopeptide; IL-6, interleukin-6; IL-10, interleukin-10; OCN, osteocalcin; OPG, osteoprotegerin; RANKL, receptor activator of nuclear factor kappa-B ligand; ROS, reactive oxygen species; TGF-β1, transforming growth factor-β 1; TRAP, tartrate-resistant acid phosphatase; Wnt/β-catenin, wingless/integrated β-catenin pathway; Pre-op, preoperation; Op., operative day; 1 day, 1 day after surgery; 3 day, 3 days after surgery; 1 week, 1 week after surgery; 1 month, 1 month after surgery; 3 month, 3 months after surgery; and 6 m, 6 months after surgery. The figure was created with BioRender software ( *https://biorender.com/* [accessed on March 5, 2026]).

**Table 2:** Summary of studies investigating changes in bone metabolic markers and serum cytokines following orthognathic surgery and surgeries at other bone sites

Study number	Subjects	Surgical intervention	Major finding	Interpretation	Ref.
1	- Patients with mandibular prognathism ( *n* = 21)	- BSSRO	- **IL-6:** increased from 1 to 3 d postoperatively. - **IL-10:** increased on the first day postoperatively. - **CRP:** increased from 1 to 3 d postoperatively. - **Neutrophil counts:** increased on the first day postoperatively. - **Neutrophil function:** increased on the first day postoperatively	Following orthognathic surgery, some patients may experience conditions like systemic inflammatory response syndrome (SIRS) and MODS (multiple-organ dysfunction syndrome). These conditions can be monitored through inflammatory cytokines and other markers	20
2	-Adult patients ( *n* = 22)	- Surgical first Le Fort I osteotomy and BSSRO	**- Orthodontic tooth movement (OTM) of maxillary and mandibular teeth:** increased from 1 wk to 3 mo postoperatively. - **ICTP:** increased from 1 wk to 3 mo postoperatively. - **ALP:** increased from 1 to 4 mo postoperatively	Orthognathic surgery induces a period of increased osteoclastic activity lasting 3–4 mo, potentially accelerating postoperative orthodontic tooth movement	19
3	- Healthy adults ( *n* = 9) for analysis of bone-related gene expression in circulating OCN+ cells - Adult patients ( *n* = 10) for determination of dynamic changes of OCN+ cells in peripheral blood	- Orthognathic surgery	**- Osteocalcin** - **positive circulating osteoblast lineage (OCN** + **cells):** increased from 1 to 4 wk postoperatively. - **Insulinlike growth factor-I (IGF-I):** decreased in the first week postoperatively and then gradually recovered. **- OCN, ALP, and COL-1 (collagen type 1) gene expression:** detected in circulating OCN cells	Circulating OCN cells could be mobilized in response to bone injury and help with bone repair in patients undergoing orthognathic surgery	22
4	-Adult patients ( *n* = 9)	- Le Fort I osteotomy of the maxilla and bilateral sagittal split of the mandible (BSSRO)	- **OPG:** continuously increased from 3 d to 6 wk postoperatively	Elevated OPG levels during the healing phase of orthognathic surgery indicate its potential as an immunological marker for monitoring postoperative bone healing and formation	24
5	- Adult patients ( *n* = 15) for serum bone metabolic markers analysis - Female New Zealand white rabbits ( *n* = 18) for micro-CT scan, changes in osteoblast and osteoclast numbers and gene expression analysis	- Le Fort I osteotomy of the maxilla, BSSRO and genioplasty. - BSSRO	- **TRAP5b:** increased from the first week to 8 wk postoperatively. - **BAP:** increased in the second week postoperatively and then gradually decreased to preoperative levels. - **Mandibular surface hollows, trabecular separation:** increased from 1 to 8 wk groups. **- Trabecular thickness, ratio of bone volume to tissue volume:** decreased from 1- to 8-wk groups. **- TRAP-positive cells (osteoclasts):** increased in the 3-wk group. - **Osteoclast:** increased from 2- to 4-wk groups. - **Osteoblast:** increased in the 3- and 4-wk groups. **- TRAP 5b gene expression:** increased from 1-wk to 3-wk groups. **- BAP gene expression and Bone morphogenetic protein 2 (BMP2) gene expression:** decreased in 1-, 2-, 3-wk groups and then increased in 4-wk group	Following orthognathic surgery, there is an increased metabolic activity in the jaw bone and a decrease in bone density, which supports the theory of RAP	21
6	Patients ( *n* = 28) (10 men and 18 women)	- Surgical first of Le Fort I osteotomy and BSSRO	- **CRP:** increased on the first day to 1 wk postoperatively. - **ICTP:** increased from 1 wk to 1 mo postoperatively. - **ALP, BAP:** decreased from the first day to 1 wk postoperatively and then increased by 1 mo postoperatively. - **OCN** + **cells:** increased from 1 d to 1 wk postoperatively	Increased inflammatory markers following orthognathic surgery indicate the presence of surgical stress. This is subsequently followed by a rise in osteoclastic activity markers, reflecting enhanced bone resorption, and later by an elevation in osteoblastic activity markers, suggesting a subsequent phase of bone formation	13
7	-Women ( *n* = 9)	- Le Fort I osteotomy and BSSRO	- **TGF-β1:** continuously increased from 3 d to 6 wk postoperatively. - **OPG:** continuously increased from 3 d to 6 wk postoperatively	Orthognathic surgery is linked to increased inflammatory cytokines and bone formation markers for up to 6 wk postoperatively. It may also affect changes in the body's immune system	23
8	-Healthy skeletal Class III men ( *n* = 16)	Le Fort I osteotomy, BSSRO and genioplasty.	**- Osteocalcin:** decreased at 3 d postoperatively. **- BAP:** decreased from 3 to 10 d postoperatively. **- ALP:** no statistically significant differences between baseline and postoperative results. **- TRAP:** decreased in 3 d postoperatively. **- Corticosterone** decreased in 3 d postoperatively. **- CRP:** increased in 3 d postoperatively. **- Inorganic:** decreased in 3 d postoperatively. **-Sodium (Na), calcium (Ca):** no statistically significant differences between baseline and postoperative results. **- Potassium (K):** decreased at 3 d postoperatively	The decrease in bone metabolic activity within the first 3 d after surgery may be a result of surgical stress	17
9	- Maxillofacial lesions patients( *n* = 48)	- Mandibulectomy, fibula-free flap, and dental implant	**- Osteocalcin:** increased from 2 to 6 mo postoperatively and then decreased by 12 mo postoperatively **- β-Cross-Laps:** decreased in 2 mo postoperatively and then increased from 4 to 6 mo postoperatively, continuously decreased to normal values by 12 mo postoperatively. **- CT data:** gradually increasing bone density over 6 mo. **- Failure of dental implant and bone marker:** correlation between low serum osteocalcin levels and elevated β-CrossLaps	Dental implants can be used for oral reconstruction following a mandibulectomy with a fibula-free flap, studies have shown good results with high long-term survival rate. OCN and β-Cross-Laps are bone metabolic markers that can monitor bone metabolism and healing. Additionally, they may serve as tools to determine the timing for implant placement	25
10	- Patients with mandibular fractures ( *n* = 20) - Healthy patients ( *n* = 15) control group	- Mandibular fractures	- **Total nitric oxide concentration, malonyldialdehyde:** increased in the mandibular fracture group both before and after the procedure. **- Nitrotyrosine:** No statistically significant differences between mandibular fracture and healthy groups. **- Asymmetric dimethylarginine:** increased in mandibular fracture group at preoperative and 1 d postoperatively	Mandibular fractures and the healing process are associated with the concentrations of NO (nitric oxide), MDA (malondialdehyde) and ADMA (asymmetric dimethylarginine). This indicates that markers of NO action in the serum can be used to assess the metabolic activity of mandibular bone. Additionally, the findings of this study may serve as a foundational principle for developing drugs that affect bone resorption by modifying NO metabolism	28
11	- 58 patients - Group 1: diaphyseal fractures of the lower leg or tibia ( *n* = 13) - Group 2: pertrochanteric femur fractures ( *n* = 10) - Group 3: femoral neck fractures ( *n* = 13) - Group 4: surgery for soft tissue diseases without bone participation (control group) ( *n* = 12) - Group 5: abdominal surgery (control group) ( *n* = 10)	- Diaphyseal fractures of the lower leg or tibia - Pertrochanteric femur fractures - Femoral neck fractures - Soft tissue surgery - Abdominal surgery	- **BAP:** decreased in the first week after surgery. - **β-CTX:** increased between days 10 and 14 postoperatively, varying by group. **- OCN:** increased between days 10 and 180 postoperatively in most groups, except for group 4, which showed no statistically significant change	Bone formation markers typically decrease initially after surgery. This is followed by an increase in bone resorption markers, and subsequently, the bone formation markers rise again. These changes reflect the processes of bone resorption and new bone formation that occur after surgery	26
12	- 57 Postmenopausal females	- Total hip arthroplasty (THA) - Total knee arthroplasty (TKA)	- **Serum OPG:** Increased after surgery then declined until month 2, peaked again at month 8, then gradually decreased. - **Serum RANKL:** increased until month 2, then declined through month 12 to below baseline levels. - **Serum osteocalcin:** decreased until day 3, then increased to a peak in month 2, and gradually decreased until month 12. - **BAP level:** decreased after surgery then increased to peak at month 8, and gradually decreased again until month 12	Metabolic bone markers show continuous changes after surgery, and these changes persist for more than 12 mo. Therefore, a 12-mo follow-up may not adequately capture the dynamic changes of metabolic bone markers following total joint arthroplasty	27

## Systemic Effects of Prediabetes on Bone Metabolic Markers

Prediabetes exerts a profound influence on bone metabolic markers and overall bone physiology, setting the stage for delayed healing and increased fracture risk. While BMD in prediabetic individuals may appear normal or even elevated, 5 6 emerging evidence underscores that bone quality is often compromised, primarily due to systemic metabolic disturbances. 5 In prediabetic and diabetic conditions, bone metabolism is significantly disrupted through multiple interconnected pathways, leading to compromised bone integrity and increased fracture risk, as illustrated in Fig. 3 .

**Fig. 3:**
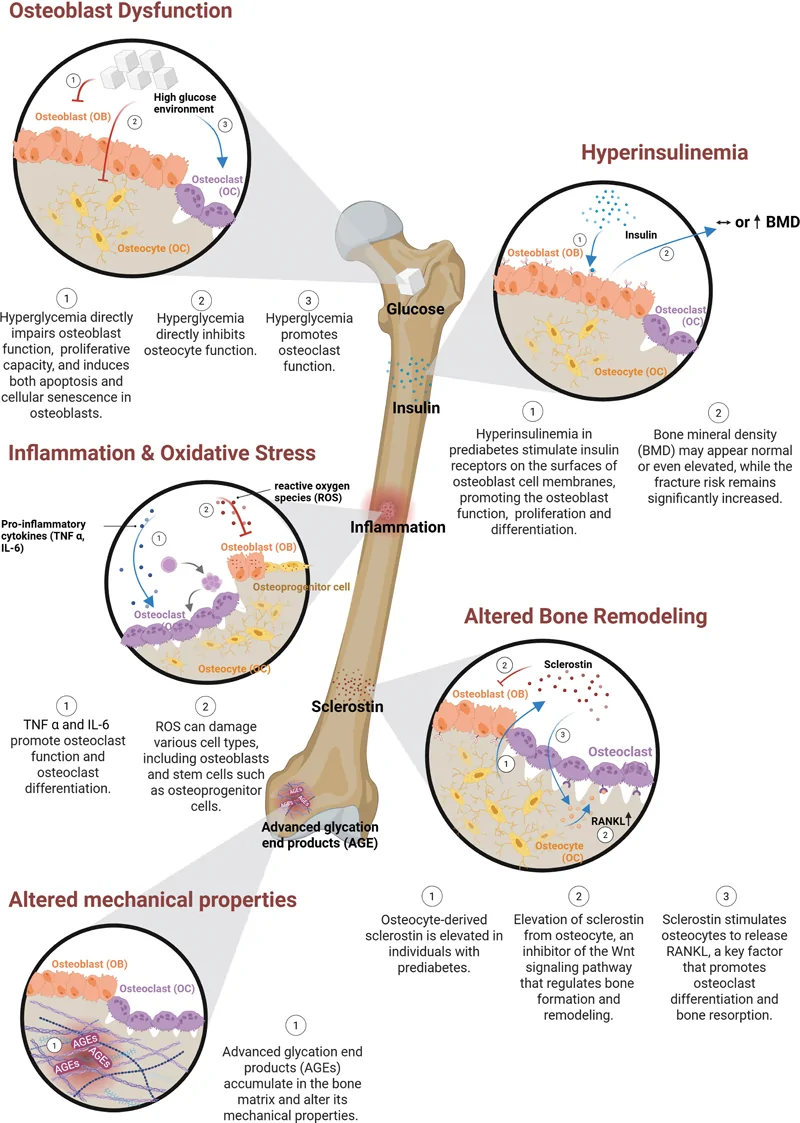
Interconnected pathways linking hyperglycemia, hyperinsulinemia, and bone metabolism in prediabetes and diabetes. Hyperglycemia impairs osteoblast function, promotes AGE accumulation, and increases osteocyte apoptosis, reducing bone quality. Hyperinsulinemia initially promotes bone formation but, over time, leads to sclerostin-mediated inhibition of the Wnt/β-catenin pathway, decreasing osteoblast activity and enhancing osteoclast function. Chronic inflammation and oxidative stress further exacerbate bone loss by promoting osteoclastogenesis and inhibiting osteoblast differentiation. The figure also highlights the role of sclerostin in altering bone remodeling, leading to increased bone resorption and reduced bone formation, ultimately compromising bone strength. BMD, bone mineral density; IL-6, interleukin-6; TNF-α, tumor necrosis factor-α; Wnt/β-catenin, wingless/integrated β-catenin pathway. The figure was created with BioRender software ( https://biorender.com/ [accessed on March 5, 2026]).

Remarkably, hyperglycemia may adversely affect osteoblast function, potentially reducing their proliferation, differentiation, and mineralization capacity. In a high-glucose environment, osteoblasts exhibit decreased matrix production and increased apoptosis, compromising bone formation. Additionally, osteocytes, the primary mechanosensory cells in bone, experience reduced viability under hyperglycemic conditions, further weakening bone strength and turnover. Mechanistically, chronic hyperglycemia in prediabetes promotes the formation of AGEs, which accumulate in the bone matrix and alter its mechanical properties. These changes weaken the structural integrity of the bone, increasing its brittleness. Additionally, AGEs disrupt osteoblast function and enhance osteocyte apoptosis, which compromises bone turnover and quality. 7

Studies also reveal that insulin resistance impairs the anabolic effects of insulin on bone, reducing osteoblast differentiation and activity. In the early stages of insulin resistance, hyperinsulinemia can paradoxically increase BMD due to the anabolic effects of insulin on osteoblasts, promoting bone formation and mineralization. Insulin stimulates osteoblast proliferation and differentiation through the insulin receptor substrate (IRS) pathway, enhancing bone matrix production and increasing BMD. However, as insulin resistance progresses, this initial anabolic response diminishes, and the protective effects on bone are lost. Prolonged hyperinsulinemia disrupts the normal balance between bone formation and resorption, leading to poor bone quality despite potentially normal or elevated BMD. This disruption is characterized by reduced osteoblast function, decreased bone turnover, and impaired mineralization, as reflected by lower levels of bone formation markers such as osteocalcin and procollagen type 1 N-terminal propeptide (P1NP). 3 A key factor in this process is the increased expression of sclerostin, a glycoprotein primarily produced by osteocytes. Sclerostin acts as a potent inhibitor of the Wnt/β-catenin signaling pathway, which is essential for osteoblast differentiation and bone formation. Elevated sclerostin levels suppress osteoblast activity, reducing bone formation and promoting osteoclast-mediated bone resorption, thereby accelerating bone loss. 4 This shift toward bone resorption, coupled with reduced osteoblast activity, results in compromised bone quality and increased fracture risk despite the apparent maintenance of BMD in some individuals with hyperinsulinemia. Additionally, low-grade chronic inflammation and elevated oxidative stress, a hallmark of prediabetes, amplify osteoclast-mediated bone resorption through elevated levels of proinflammatory cytokines like interleukin-6 (IL-6) and tumor necrosis factor-α (TNF-α). These cytokines not only promote osteoclastic activity but also inhibit osteoblast differentiation, tipping the balance toward bone resorption. 32 33 Reactive oxygen species (ROS), which are elevated in hyperglycemic conditions, directly damage osteoblasts and osteocytes, further reducing bone formation and increasing resorption. 7 This inflammatory environment not only promotes osteoclastogenesis but also impairs osteocyte function, tipping the balance toward bone resorption. 34 Population-based studies have reported that individuals with high prediabetes and greater insulin resistance exhibit significantly lower trabecular bone score (TBS), indicating deterioration in trabecular microarchitecture despite preserved BMD. 35 Alterations in bone metabolic markers in the context of prediabetes have been extensively studied due to their critical role in bone turnover and remodeling under metabolic disturbances. A summary of the functions and observed changes in key bone markers in prediabetic individuals compared with healthy controls is provided in Table 3 .

**Table 3:** Summary of bone metabolic markers and their alterations in prediabetes

Bone metabolic markers	Function	Source	Sample	Methods	Prediabetes	Ref.
CTX	Bone resorption	Bone and other tissues containing type I collagen	Serum, plasma, urine	ELISA RIA ECLIA	Decreased or similar to healthy controls	3 16 42 54 55 56
uNTX	Bone resorption	Bone and other tissues containing type I collagen	Urine	ELISA CLIA RIA	- (Not associated with fasting plasma glucose)	54 55 56 57
TRAP	Bone resorption	Osteoclasts	Serum, plasma	Colorimetry RIA ELISA CLIA	Increased	36 54 55 56
FGF-23	Bone resorption	Osteocytes	Plasma	ELISA CLIA	Increased	37 54 55 56
Sclerostin	Bone resorption	Osteocytes, chondrocytes	Serum, plasma	ELISA CLIA	Increased	4 54 55 56
ALP	Bone formation	Bone, liver, kidney	Serum, plasma	Electrophoresis Colorimetry Precipitation CLIA IRMA EIA	Decreased	39 54 55 56
BAP	Bone formation	Osteoblasts	Serum, plasma	Electrophoresis Precipitation IRMA EIA HPLC	Increased or decreased	38 54 55 56
OCN	Bone formation	Osteoblast, platelets, dentine, hypertrophic cartilage	Serum, plasma, urine	RIA IRMA ELISA CLIA ECLIA	Decreased or similar to healthy controls	16 44 54 55 56 57
P1NP	Bone formation	Bone and other tissues containing type I collagen	Serum, plasma	RIA ELISA CLIA	Decreased or similar to healthy controls	3 16 42 44 54 55 56
OPG	Bone formation	Osteoblast	Serum, plasma	ELISA	Increased	41 54 55 56 58

Emerging evidence underscores the relationship between glycemic dysregulation and impaired bone turnover. A recent study demonstrated a negative correlation between bone turnover markers—specifically serum C-terminal telopeptide of type I collagen (s-CTX) and procollagen type 1 N-terminal propeptide (s-P1NP)—and glycemic variability, indicating that greater fluctuations in blood glucose levels are associated with reduced bone remodeling activity. Notably, such metabolic disturbances are not confined to overt diabetes but are also observed in the prediabetic state, where IFG and insulin resistance begin to adversely affect bone metabolism. 3 Additionally, levels of C-terminal telopeptide of type I collagen (CTX), a key indicator of bone resorption, are reported to be either decreased or similar to those in healthy controls among individuals with prediabetes. 3 16 However, urinary N-telopeptide (uNTX), another marker reflecting resorptive activity, has been studied but shows inconclusive trends in prediabetic populations. Elevated levels of TRAP indicate enhanced osteoclastic activity in prediabetes, contributing to increased bone resorption. 36 Similarly, fibroblast growth factor-23 (FGF-23), a marker of bone resorption, is notably elevated, reflecting further disruptions in bone turnover. 37 Additionally, sclerostin, an inhibitor of the Wnt signaling pathway that regulates bone formation and remodeling, is significantly elevated in prediabetes, underscoring a shift toward impaired bone metabolism. 4 In terms of bone formation, alkaline phosphatase (ALP), a marker of osteoblastic activity, is consistently decreased in prediabetic individuals, signaling reduced bone formation. Bone-specific alkaline phosphatase (BAP), although closely linked to bone formation, shows less clearly defined trends in prediabetes. 38 39 40 Osteocalcin, another key bone formation marker, demonstrates levels that are decreased or similar to those in healthy controls, reflecting variability in osteoblast function. 16 Procollagen type I N-terminal propeptide (P1NP), a vital marker of new bone synthesis, is also reported to exhibit decreased or comparable levels, indicating inconsistent effects on bone formation. 3 16 Conversely, elevated levels of OPG in prediabetes suggest a compensatory response to counteract increased bone resorption. 41

Various studies provide strong evidence that metabolic disturbances associated with prediabetes and diabetes significantly impact bone turnover, BMD, and healing potential. These findings are particularly relevant in maxillofacial and orthognathic surgeries, where bone health is crucial for postoperative recovery. A summary of studies investigating the effects of prediabetes and dysglycemia on bone cells and bone metabolism is presented in Table 4 . In terms of bone resorption and its link to glucose dysregulation, CTX exhibits a **U** -shaped relationship with glucose metabolism. 34 Individuals with excessively high or low CTX levels were more likely to progress from normal glucose tolerance (NGT) to prediabetes, whereas those with moderate CTX levels exhibited the lowest risk. 34 This suggests that bone remodeling is dynamically linked to glucose regulation, and an imbalance in bone resorption may either reflect or contribute to metabolic dysfunction. These findings align with the hypothesis that bone acts as an endocrine organ, modulating systemic glucose homeostasis through bone-derived factors such as osteocalcin (OCN). 16

**Table 4:** Summary of studies on prediabetes-associated bone metabolism and therapeutic strategies targeting bone healing and homeostasis

Study number	Subjects	Intervention	Major finding	Interpretation	Ref.
1	- Normal glucose tolerant (NGT) women ( *n* = 195)	- Longitudinal 4 y follow-up	- Demonstrated a U-shaped relationship between serum CTX levels and incident dysglycemia	The relationship between serum CTX levels and the occurrence of dysglycemia seems to follow a U-shaped pattern. Mildly increased osteoclast activity may provide a protective, compensatory effect during the early stages of glucose dysregulation. However, excessive bone resorption can lead to skeletal fatigue and a reduced ability to maintain glucose homeostasis	34
2	- Maxillofacial surgery patients ( *n* = 1,374)	Retrospective analysis of patients' electronic health records	- Increased the incident of infection and necrosis in patients with max blood glucose >200 mg/dL compared with patients without a pathological glucose metabolism. - Increased duration of inpatient treatment in known diabetes, impaired fasting glucose, and high maximum blood glucose	The presence of diabetes does not necessarily correlate with an increased incidence of complication of maxillofacial surgical procedures. Furthermore, individuals with well-controlled diabetes tend to have comparable surgical outcomes non-diabetes patients. However, having hyperglycemia has an significant effect on treatment outcomes	8
3	- Normoglycemic men ( *n* = 643) - Normoglycemic women ( *n* = 682) - Impaired fasting glucose men ( *n* = 355) - Impaired fasting glucose women ( *n* = 391) - Diabetic men ( *n* = 97) - Diabetic women ( *n* = 54)	- Blood sample for serum glucose and bone metabolic markers (CTX and P1NP) analysis - Dual energy X-ray absorptiometry (DXA) for femoral neck BMD measurement	- **CTX and P1NP:** decreased in prediabetic and diabetic individuals compared with normoglycemic individuals. **- BMD:** decreased in prediabetic and diabetic individuals compared with normoglycemic individuals	Individuals with prediabetes already exhibit suppressed bone turnover, similar to those with diabetes. These findings suggest that altered bone remodeling begins before the onset of clinical diabetes, emphasizing the importance of early metabolic monitoring	42
4	- Healthy normoglycemic individuals ( *n* = 43) - Prediabetic individuals ( *n* = 79)	- Oral glucose tolerance test (OGTT) - Euglycemic -hyperinsulinemia clamp with tracer - Blood sample for serum glucose and sclerostin analysis - Dual energy X-ray absorptiometry (DXA) for determining fat and lean body mass and whole-body minus head bone mineral density	- Serum sclerostin was significantly elevated in prediabetes and positively correlated with HOMA-IR and HbA1c. - Negative correlation between serum sclerostin with insulin clearance	Sclerostin levels are higher in prediabetic individuals, particularly those with impaired fasting glucose and insulin resistance. This suggests that sclerostin may play a role in the development of glucose intolerance, linking bone health to metabolic health	4
5	- Chinese postmenopausal women ( *n* = 271) - T2DM group ( *n* = 93) - Prediabetes group ( *n* = 90) - Normoglycemic group ( *n* = 88)	- Blood sample for FPG, 2hPG, HbA _1c,_ ALT, AST, ALP, BUN, Cr, HDL-C, LDL-C, total cholesterol (TC), triglycerides (TG), and blood uric acid (URCA) measurements - Homeostasis model assessment for insulin resistance analysis (HOMA-IR)	- **OPG level:** increased in prediabetes and T2DM groups. **- OPG level** was positively correlated with HOMA-IR, 2hPG, HbA1c, waist-to-hip ratio, body fat percentage, ALP and AST **- OPG level** was negatively correlated with lumbar spine BMD, femoral neck BMD and eGFR	Serum OPG levels are elevated in postmenopausal women with prediabetes and type 2 diabetes, correlating with insulin resistance. OPG may serve as an early biomarker linking bone remodeling to metabolic and vascular dysfunction, highlighting the bone–vascular–metabolic axis in diabetes pathophysiology	41
6	**- Sedentary healthy group (** ***n*** ** = 28)** - SIT group ( *n* = 14) - MICT group ( *n* = 14) **- Insulin resistant (IR) group (diabetes and prediabetes) (** ***n*** ** = 26)** - SIT group ( *n* = 13) - MICT group ( *n* = 13)	- Sprint interval training (SIT) - Moderate intensity continuous training (MICT)	**After the training intervention:** **- Whole-body insulin sensitivity:** improved in both groups. **- P1NP and osteocalcin:** still decreased in IR group. **- Lipid and glucose profiles:** improved in both groups. **- Insulin-stimulated glucose uptake in femoral bone marrow:** improved in both groups.	Bone metabolism and turnover rates are lower in individuals with insulin resistance (IR). Osteocalcin and PINP correlated positively with femoral bone marrow glucose uptake and glycemic status.	44
7	Postmenopausal female patients with T2DM and naive to insulin and incretin based treatments ( *n* = 30) - Exenatide - Insulin glargine	- Exenatide (glucagon-like peptide-1 receptor analogs) - Insulin glargine (injectable long-acting insulin)	- **RANK, RANKL and BMI:** decreased in exenatide group. - **OPG** : increased in exenatide group. - **ALP, BAP:** no statistically significant differences in both groups - **NTX:** no statistically significant differences in both groups - **BMD:** no statistically significant differences in both groups **- HbA1c:** improved in both groups	Exenatide significantly increased OPG and decreased bone resorption markers (RANK, RANKL), indicating a potential anti-resorptive effect via the OPG/RANK/RANKL pathway. However, bone formation markers and BMD were unchanged, suggesting neither treatment significantly affected overall bone formation.	48
8	T2DM patients ( *n* = 371)	- Insulin aspart (short acting insulin) - NPH insulin (long-acting insulin) - Rosiglitazone - Placebo	- **CTX:** decreased in 3 months then increased in 12 months and 24 months. No difference between short-acting (aspart) and long-acting (NPH) insulin. - **P1NP:** unchanged in 3 mo and significantly increased in 12 and 24 mo. No difference between short-acting (aspart) and long-acting (NPH) insulin. - **HbA1c:** negative correlation between HbA1c and CTX	The selection of insulin therapy does not impact bone metabolic markers; however, the administration of metformin may reduce BTMs, and improved glycemic control correlated with increased bone resorption, potentially reflecting restoration of physiological remodeling in T2DM's low-turnover skeletal phenotype.	49
9.	- T2DM and naive to oral hypoglycemic drugs patient in A Diabetes Outcome Progression Trial (ADOPT) ( *n* = 1605)	- Rosiglitazone - Metformin - Glyburide	**Men** **- Parathormone (PTH):** decreased in rosiglitazone and metformin groups compared with baseline. **- Estradiol:** decreased in rosiglitazone group compared with baseline. **- CTX:** decreased in metformin group compared with baseline. **- P1NP:** decreased in metformin and rosiglitazone groups compared with baseline. **- BAP:** decreased in all groups compared with baseline. **Women** **- PTH:** decreased in rosiglitazone and metformin groups. compared with baseline. **- Estradiol:** decreased in rosiglitazone group compared with baseline. **- CTX:** no statistically significant differences between baseline and postoperative results. **- P1NP:** decreased in metformin group compared with baseline. **- BAP:** decreased in all groups compared with baseline.	Rosiglitazone increased bone resorption in women, shown by elevated CTX levels, while metformin and glyburide had neutral effects. In men, metformin significantly reduced bone resorption, whereas rosiglitazone and glyburide had little impact. These findings suggest thiazolidinediones like rosiglitazone increases bone resorption markers in women, potentially contributing to higher fracture risk.	50
10.	- Elderly men with type 2 diabetes mellitus ( *n* = 120)	- Control group (low-dose metformin: 0.5 g × 2/day) ( *n* = 60) - Experimental group (high-dose metformin: 0.5 g × 4/day) ( *n* = 60)	- **Glycemic control:** no statistically significant differences between groups. - **BMD:** higher in experimental group. - **25(OH)D:** higher in experimental group. - **Osteocalcin:** lower in experimental group. - **β-CTX:** lower in experimental group	Both low and high doses of metformin improve blood sugar control in elderly men with type 2 diabetes. A higher dose yields greater benefits for bone density and metabolism, suggesting it supports skeletal health alongside glucose regulation. However, long-term high-dose use may impair bone formation, requiring further research to find the optimal balance between metabolic benefits and bone health	51
11.	- Obese patient without diabetes ( *n* = 195)	After 8 wk on an 800 kcal/day diet, participants were randomized to 1 of 4 groups for 52 wk: 1. Exercise alone 2. Liraglutide alone 3. Liraglutide + exercise 4. Placebo + no exercise	**After an 8-wk of low-calorie diet** - **Body lean mass** : increased in placebo and exercise groups. Combination and liraglutide preserved lean mass. - **BMD:** decreased in liraglutide group compared with placebo. - **CTX** : increased after an 8-week of low-calorie diet and decreased by week 26 across all groups, with the placebo group showing the lowest levels. - **P1NP:** increased after an 8-week of low-calorie diet and then gradually returned to preoperative levels in all groups by week 52	Exercise combined with GLP-1RA (liraglutide) effectively promotes weight loss while preserving bone health, unlike liraglutide alone, which resulted in a reduction of both body weight and (BMD). This indicates that exercise is crucial for maintaining bone health during weight loss, likely due to its mechanical effects on bone remodeling and preservation of lean mass. These findings emphasize the importance of integrating exercise with pharmacological obesity treatments like GLP-1RAs to prevent bone loss and support long-term skeletal health in weight management strategies	52
12.	- Women with prediabetes and hypovitaminosis D ( *n* = 168)	- Placebo - Omega-3 supplements - Vitamin D supplements - Omega-3 and vitamin D co-supplementation	**- Fasting blood glucose (FBS), fasting insulin,** and **HOMA-IR (insulin resistance index):** decreased in the co-supplementation group. **- HOMA-B (β-cell function):** improved in the co-supplementation group	Eight weeks of vitamin D and omega-3 co-supplementation significantly improved fasting glucose, insulin sensitivity, HDL cholesterol, weight, and waist circumference in prediabetic women with hypovitaminosis D. These findings support the potential role of omega-3 and vitamin D as supplementary therapies to enhance bone healing after surgery and prevent metabolic bone disorders in at-risk groups	53

Importantly, a study investigating how diabetes and hyperglycemia influence maxillofacial surgical outcomes demonstrated that while well-controlled diabetes did not significantly increase the risk of surgical complications, patients with elevated blood glucose levels (≥200 mg/dL) had a higher incidence of infection and necrosis, along with prolonged inpatient stays. 8 These findings reinforce the importance of strict perioperative glycemic control in diabetic patients undergoing maxillofacial surgery. It also suggests that hyperglycemia rather than diabetes itself may be the key determinant of surgical complications, emphasizing the need for real-time glucose monitoring and regulation in surgical patients. Additionally, several studies confirmed that bone metabolism is disrupted in prediabetes and diabetes, leading to reduced BMD and alterations in bone turnover markers. Holloway-Kew et al 42 and Li et al (2022) 43 demonstrated that both men and women with IFG and diabetes had lower CTX and procollagen type 1 N-terminal propeptide (P1NP) levels compared with normoglycemic individuals. This suggests that bone formation and resorption are both impaired in glucose dysregulation, potentially increasing the risk of fractures and delayed healing following surgery. Since bone turnover decreases even before diabetes develops, individuals with prediabetes may already be at risk for compromised bone health, which is an important consideration for orthognathic and maxillofacial surgery candidates. Consistently, sclerostin—a Wnt signaling inhibitor that suppresses bone formation—was elevated in individuals with prediabetes and correlated with insulin resistance. 4 Moreover, OPG, a regulator of bone resorption, was significantly elevated in postmenopausal women with prediabetes and diabetes, suggesting that elevated OPG levels were associated with higher insulin resistance (IR). 41 These findings imply that targeting bone-derived proteins like sclerostin and OPG could be an innovative strategy to mitigate both bone loss and metabolic dysfunction in prediabetic and diabetic patients. Additionally, A study using PET imaging demonstrated that insulin-stimulated glucose uptake in femoral bone marrow was reduced in IR patients, suggesting a decline in bone marrow metabolic activity. They also found that OCN and PINP correlated positively with femoral bone marrow glucose uptake and glycemic status. 44 Collectively, prediabetes and diabetes not only impair bone turnover and mineral density but also influence key bone regulatory proteins such as CTX, P1NP, sclerostin, and OPG. These alterations may have profound implications for bone healing, surgical outcomes, and fracture risk. Understanding these relationships is critical for optimizing patient management in orthognathic surgery and other bone-related procedures. Screening for key bone metabolic markers could help identify patients at risk for bone fragility and delayed healing after surgery.

## Therapeutic Targets for Bone Homeostasis and Their Implications for Bone Healing after Orthognathic Surgery under Prediabetic or Hyperglycemic Conditions

Cumulative evidence from current studies suggests that optimizing metabolic health through lifestyle modifications, pharmacologic treatments, nutritional interventions, and exercise may significantly improve bone healing outcomes in prediabetic individuals undergoing surgery. Since bone remodeling is impaired in prediabetes and diabetes, implementing these strategies preoperatively may enhance bone regeneration, reduce fracture risk, and accelerate postsurgical healing. Similar to other invasive procedures, orthognathic surgery can significantly impact a patient's quality of life due to its complex recovery process, which may be further complicated in individuals with metabolic disturbances such as prediabetes. Patient satisfaction and functional outcomes after orthognathic procedures are sensitive to healing quality and complication rates. 45 Despite the growing recognition of the impact of diabetes on bone health, studies focusing on prediabetes and dysglycemia remain sparse. Emerging evidence suggests that targeting molecular pathways involved in bone metabolism may offer promising therapeutic strategies for improving bone healing in these conditions. Understanding how therapeutic interventions can modulate bone homeostasis in prediabetic and hyperglycemic states could provide valuable insights into optimizing surgical outcomes in affected patients. A summary of studies involving therapeutic targets for bone homeostasis and their implications for bone healing after orthognathic surgery under prediabetic or hyperglycemic conditions in Table 4 .

Two independent studies on lifestyle modifications and prevention of dysglycemia-related bone complications demonstrated that diet and exercise interventions significantly reduce the incidence of diabetes among individuals with IGT. 46 47 Both studies showed that targeted lifestyle modifications effectively delay or prevent the transition from prediabetes to type 2 diabetes (T2DM), reinforcing the importance of early intervention in high-risk individuals. Given that glucose dysregulation negatively affects bone remodeling, these findings suggest that maintaining glycemic stability through lifestyle modifications could mitigate bone healing deficits in prediabetic individuals undergoing maxillofacial or orthognathic surgery. Among these, incretin-based therapies (GLP-1 receptor agonists like exenatide) exhibit bone-protective effects by enhancing OPG levels and reducing bone resorption markers (RANK and RANKL), suggesting a potential inhibition of osteoclast activity. However, despite these positive changes, bone formation markers and BMD remained unchanged, indicating that GLP-1 receptor agonists primarily prevent bone breakdown rather than promote bone formation. 48 This highlights the necessity for additional interventions to enhance bone anabolism alongside their anti-resorptive effects. Conversely, insulin therapy alone does not appear to significantly influence bone turnover markers, as neither short-acting nor long-acting insulin showed a statistically significant effect on bone metabolism markers such as CTX or P1NP. 49 However, the addition of metformin to insulin therapy was associated with reduced bone turnover markers, suggesting that metformin plays a role in suppressing excessive bone resorption. This finding aligns with previous research, where metformin was shown to lower both CTX and P1NP levels, thereby reducing both bone resorption and formation. 50 A major concern arises with thiazolidinediones (TZDs) such as rosiglitazone, which were associated with increased bone resorption (elevated CTX) in women and a reduction in bone formation markers (P1NP and BAP) in both men and women. 50 These findings reinforce concerns that TZDs contribute to bone loss and increased fracture risk, particularly in postmenopausal women. Moreover, rosiglitazone-induced reductions in estrogen levels may exacerbate bone deterioration, making its use in women with T2DM particularly concerning. Interestingly, higher doses of metformin were associated with improved BMD, suggesting a dose-dependent effect on bone mineralization. However, excessive suppression of bone turnover could potentially impair bone remodeling, emphasizing the need for careful dose considerations, particularly in elderly patients. 51

Beyond pharmacological interventions, lifestyle modifications and nutritional supplementation are increasingly recognized for their crucial role in maintaining bone health. Exercise has long been established as a key factor in promoting bone strength, and a previous study confirmed that combining GLP-1 receptor agonists with exercise can mitigate the bone loss typically associated with weight loss therapies. Conversely, liraglutide alone led to BMD reduction, reinforcing the idea that mechanical loading through exercise is necessary to maintain bone integrity. 52 These findings emphasize the importance of integrating physical activity with pharmacological interventions to prevent bone loss in obese and diabetic individuals undergoing weight management programs. 52 Similarly, another study highlighted the negative effects of insulin resistance on bone marrow metabolism, revealing reduced osteocalcin and P1NP levels in individuals with IGT. While short-term exercise training improved glucose and fatty acid metabolism in bone marrow, it did not significantly enhance bone metabolic markers, suggesting that while exercise aids systemic metabolism, its direct effects on bone formation require longer-term interventions. 44 A particularly promising avenue for bone health interventions is the co-supplementation of omega-3 and vitamin D, as demonstrated in another study. This intervention significantly improved glycemic control, lipid profiles, and inflammatory markers in prediabetic women. Since vitamin D is essential for calcium absorption and osteoblast function, supplementation likely contributed to enhanced bone mineralization. Moreover, omega-3 fatty acids possess well-documented anti-inflammatory properties, which may counteract chronic inflammation-driven bone loss commonly observed in prediabetic individuals. 53 Collectively, these studies underscore the need for a multifaceted approach to preserving bone health in individuals with T2DM and prediabetes, integrating pharmacological, lifestyle, and nutritional interventions. Given the negative impact of TZDs on bone metabolism, alternative therapies such as GLP-1 receptor agonists (exenatide, liraglutide) and metformin should be prioritized for diabetic patients at risk of osteoporosis. Additionally, combining exercise with pharmacological weight loss therapies may be crucial in preventing BMD decline, particularly in obese individuals undergoing metabolic treatments. Lastly, nutritional supplementation with omega-3 and vitamin D represents a promising adjunctive strategy for improving both metabolic health and bone remodeling, particularly in prediabetic individuals prone to bone fragility. These findings indicate that integrative treatment strategies combining pharmacotherapy, lifestyle changes, and nutritional support hold theoretical promise for optimizing bone health. However, concrete, evidence-based protocols for perioperative metabolic management are currently lacking. Therefore, prospective clinical trials are highly warranted rather than providing prescriptive clinical guidance at this stage. 53

## Limitations of the Current Evidence

While the synthesized data provide valuable insights, several limitations in the current literature must be acknowledged. First, there is significant heterogeneity in the biomarker assays utilized across studies (e.g., ELISA, ECLIA, RIA), which complicates direct comparisons of marker concentrations. Second, the temporal sampling windows for bone turnover markers vary widely among studies, making it challenging to establish a standardized kinetic timeline for postoperative healing. Third, many studies lack robust statistical adjustment for key confounding variables, such as patient age, sex, menopausal status, BMI, and concurrent medications. Finally, much of the mechanistic data are derived from in vitro or animal models, and there is a distinct lack of large-scale, prospective clinical trials specifically evaluating orthognathic surgery outcomes in prediabetic populations.

## Conclusion

Bone remodeling is tightly regulated and influenced by systemic conditions like prediabetes, which negatively impacts bone healing and metabolic markers. This has significant implications for orthognathic surgery outcomes. Hyperglycemia is associated with impaired bone healing, suggesting potential for damage to bone tissue and a disruption in the balance of osteoblasts and osteoclasts. This imbalance, driven by AGEs, chronic inflammation, and insulin resistance, increases the risk of delayed healing and surgical complications. Additionally, the systemic acceleratory phenomenon (SAP) may be impaired in prediabetic patients, resulting in suboptimal recovery. This highlights the potential value of exploring comprehensive approaches to perioperative management in these individuals.

Although surgical interventions remain the gold standard for correcting skeletal deformities, the potential role of metabolic management in optimizing bone healing represents a critical area for future investigation. Currently, evidence-based clinical protocols do not exist. Nevertheless, current evidence suggests that glycemic control through lifestyle modifications, pharmacological interventions (such as GLP-1 receptor agonists and metformin), and nutritional supplementation may significantly improve bone regeneration. Furthermore, integrating physical exercise is crucial to mitigate the negative effects of weight loss therapies on BMD. Developing novel interventions should target underlying mechanisms. Since chronic inflammation and oxidative stress impair remodeling, future research should explore anti-inflammatory and bone anabolic therapies. Additionally, the role of bone-derived hormones, such as osteocalcin and sclerostin, warrants investigation to determine their potential as therapeutic biomarkers.

To optimize clinical outcomes, future studies must prioritize well-designed, prospective clinical trials to validate the efficacy of these interventions in prediabetic orthognathic patients. Longitudinal studies on the long-term effects of metabolic disturbances are also needed. Moreover, integrating personalized medicine and biomarker-based screening could enable the early identification of at-risk patients, allowing for tailored interventions to improve bone.
